# Transformations of Diatom-Derived Dissolved Organic Matter by *Bacillus pumilus* Under Warming and Acidification Conditions

**DOI:** 10.3389/fmicb.2022.833670

**Published:** 2022-02-25

**Authors:** Yang Liu, Xueru Wang, Jun Sun

**Affiliations:** ^1^Institute of Marine Science and Technology, Shandong University, Qingdao, China; ^2^Research Center for Indian Ocean Ecosystem, Tianjin University of Science and Technology, Tianjin, China; ^3^College of Marine Science and Technology, China University of Geosciences, Wuhan, China; ^4^State Key Laboratory of Biogeology and Environmental Geology, China University of Geosciences, Wuhan, China

**Keywords:** *Skeletonema dohrnii*-derived DOM, *Bacillus pumilus*, EEM-PARAFAC, fluorophores, spectroscopic indices

## Abstract

Heterotrophic bacteria are assumed to play an important role in processing of phytoplankton-derived dissolved organic matter (DOM). Although the algae-derived organic matter is commonly studied, the transformation and processing of DOM by epiphytic bacteria for phytoplankton have rarely been investigated, especially under warming and acidification. In this study, *Bacillus pumilus* is used to explore the ecologically important marine diatom *Skeletonema dohrnii*-derived DOM under different conditions (temperature, 27°C and 31°C; *p*CO_2_, 400 and 1,000 ppm), utilizing fluorescence excitation-emission matrix (EEM) combined with parallel factor analysis (EEM-PARAFAC). Fluorescence regional integration and the peak selecting method are used to generate B, T, N, A, M, and C peaks in the EEM fluorescence spectroscopy. The main known fluorophores including that protein-like components (peaks B and T), unknown components (peak N), and humic-like component (peaks A, M, and C). Our experimental results showed that under higher temperature and pressure of CO_2_ (*p*CO_2_) conditions, *S. dohrnii*-derived DOM fluorescence was dominated by a protein-like signal that slower waning throughout the experiment, becoming an increasingly humic-like substance, implying that processing by the epiphytic bacteria (*B. pumilus*) produced more complex molecules. In addition, spectroscopic indices (e.g., fluorescence index, biological index, freshness index *β/α*, and humification index) were changed in varying degrees. This study reveals and confirms the direct participation of heterotrophic bacteria in the transformation and generation of algae-derived DOM in the laboratory, underlining the influence of global warming and ocean acidification on this process.

## Introduction

The primary type of organic matter in the oceanic environment is phytoplankton-derived dissolved organic matter (DOM), which contributes to the world’s greatest carbon pool (662 Pg C) (Hansell et al., 2009, 2012). Diatoms, one of the most abundant and diverse groups of marine phytoplankton, contributing ∼20% of global primary productivity (Thangaraj and Sun, 2021). At a rate of 43 Tg per year, more than 90% of dissolved organic carbon (DOC) could be generated, playing an essential role in the global carbon cycle (Thornton, 2014). The vast majority of DOC is changed, resulting in recalcitrant DOC, which is then exported to the deep sea (Hansell et al., 2012).

For almost 200 million years, algae and bacteria have coexisted in aquatic environments (Falkowski et al., 2004). Heterotrophic bacteria are key biogeochemical regulators in aquatic systems. The majority of the DOM metabolism produced by algae enters the microbial cycle, where it provides carbon and nutrients to heterotrophic bacteria (Pomeroy et al., 2007). Meanwhile, the bioreactivity and chemical composition of DOM have changed during/after microbial utilization (Liu et al., 2021a). Heterotrophic bacteria link biogeochemical cycles together by decomposing and producing organic matter. Most organic carbon fixed by photosynthesis is consumed by heterotrophic bacteria (Del Giorgio and Duarte, 2002). According to one study, increased *p*CO_2_ will enhance vertical carbon flux (Siu et al., 2014), while another suggested that bacteria with high *p*CO_2_ and temperature conditions have increased rates of polysaccharide degradation, protein production and enzymatic activity (Grossart et al., 2006). In brief, marine bacterioplankton play an important part in the carbon cycle. On the other hand, carbon consumption and transport rates of heterotrophic bacteria govern the efficacy of carbon sequestration in the ocean (Fuhrman, 2009).

The temperature and CO_2_ concentrations have increased rapidly since the onset of the industrial revolution, led to global climate change (Meehl et al., 2007). Seawater, on the other hand, is becoming progressively acidified due to oceanic absorption of atmospheric CO_2_ (Meehl et al., 2007; Pachauri et al., 2014). Marine ecosystems are particularly sensitive to environmental changes, as species are stressed by both warming and ocean acidification (Riebesell et al., 2018). The mean sea surface temperatures are forecast to rise by 4°C by the end of the twenty-first century, while CO_2_ levels in the atmosphere are expected to treble (Meehl et al., 2007). The creatures and processes in the seas will surely suffer as a result of these cumulative impacts (Thangaraj and Sun, 2021). Ocean acidification and warming are seen as severe threats to the marine species (Stocker et al., 2014). Previous studies reported that ocean warming and acidification affect the physiological and biochemical state of diatoms (Thangaraj and Sun, 2020), but also have an impact on their photosynthesis and metabolism (Thangaraj and Sun, 2021). The potential response of the algae varies to the extent that the effect of bacterial utilization and transformation of algae-derived organic matter is uncertain. However, very few snapshots are available on the use of algae-derived organic matter by unibacteria under warming and acidification, let alone the combined effects of both factors.

Excitation-emission matrix (EEM) fluorescence spectroscopy is prevalent approaches for analyzing organic matter due to the vast quantity of visual maps, and three-dimensional information it gives (Rodríguez-Vidal et al., 2020). Recently, EEM spectroscopy combined with parallel factor analysis (PARAFAC) have been frequently employed to the characterization of DOM due to its remarkable sensitivity and selectivity (Lin et al., 2021; Liu et al., 2021d). Protein- and humic-like fluorophores have been found in previous studies, and their peak locations in EEM spectroscopy make it straightforward to distinguish between them (Coble, 1996; Coble et al., 1998).

In this study, we used single bacteria (*B. pumilus*) to investigate the role and preference for *S. dohrnii*-derived DOM under warming and acidification conditions. Our research builds upon laboratory-based studies over the 30-day timespan. We examined the characteristics of fluorescent organic matter by using EEM-PARAFAC methods. In addition, using different parameters (e.g., bacteria abundance, DOC, pH, fluorescence indices, peaks, and components) to further characterize the variation in organic matter *via* the processing of different bacterial under warming and acidification. We hypothesized that the DOM characteristics should be significantly correlated with bacterial utilization and transformation, and that they would exhibit different patterns.

## Materials and Methods

### *Skeletonema dohrnii* Culture Conditions

Marine diatoms (e.g., *Skeletonema* spp.) are widely distributed in offshore China, especially, *S. dohrnii* have been found and isolated in China’s Yellow Sea coastal waters, and subsequently preserved in our laboratory. *S. dohrnii* cells were pre-cultivated and transferred in artificial seawater (ASW) medium. The cultivation was conducted at 25°C with a light intensity of 100 μmol photons m^–2^ s^–1^ and a 14:10 h light:dark cycle. The cells were grown at least for three generations before the initiation of the experiment.

### Isolation and Identification of Epiphytic Bacteria

Epiphytic bacteria was isolated from the degradation growth stage of microalgae by gradient dilution method. The 2216E agar plates were used to isolate epiphytic bacteria, which cultured in transparent conical flasks (500 mL) in a shaking incubator (26°C, 150 rpm). To identify the epiphytic bacteria, the genomic DNA of bacteria was extracted by TIANamp Bacteria DNA Kit (Tiangen-Biotech, Beijing, China). For polymerase chain reaction (PCR) amplification of the 16S rDNA V3 region, a universal bacterial primer was used. The phylogenetic tree (Neighbor-Joining tree, N-J tree) was constructed using the bacteria sequences and closest related sequences from GenBank, and the genetic distances were calculated. Based on the results of analysis, the isolated bacterial strains (CA-35) shared 66.67% sequence identity to the valid species (*Bacillus pumilus*) from GenBank.

### Biochemical Characterization of Bacteria

The isolated positive colonies were identified by Gram staining reaction and biochemical tests (including arabinose test, glucose test, maltose test, urease test, Vogues Proskauer test, nitrate reduction test, starch hydrolysis test, aesculin test, 7% NaCl test, and pH 5.7 test).

### Flow Cytometry of *B. pumilus* Abundance

The strains *B. pumilus* was cultured in 500 mL transparent conical flasks with pre-combusted (450°C, 5 h). As described in previous studies (Moens et al., 2016), the abundance of *B. pumilus* cells was measured by Accuri C6 flow cytometer (BD Biosciences, Erembodegem, Belgium). 0.01% SYBR Green I was applied to the sample to stain it for 30 min in the dark at 37°C. As an internal standard, 1 μm fluorescent beads (Polyscience, Warrington, PA, United States) were injected into each sample. Then, the samples were measured at a flow rate of 0.25 μL s^–1^ for 1 min.

### Measurements of Dissolved Organic Carbon and pH

To avoid any carbon contamination, all glass materials were acid washed, rinsed with ultra-pure water, and precombusted (450°C for 5 h). A total organic carbon analyzer (TOC-3100, Germany) was used to detect the dissolved organic carbon (DOC). All DOC samples were gravity filtered using the GF/F glass fiber filters (0.7 μm pore size, 47 mm diameter, Whatman). GF/F glass fiber filters can be cleaned by high temperature combustion and can filter sufficient sample volumes without clogging, thus reducing potential sources of contamination. The pH variation of culture was measured using pH meter (Lab 850, SCHOTT Instruments).

### *Skeletonema dohrnii*-Derived Dissolved Organic Matter Collection and Experimental Setup

*Skeletonema dohrnii* cells were cultivated in ASW medium, and after reaching the degradation growth phase [algal concentration was around (4.07 ± 0.02) × 10^7^ cells L^–1^]. Microalgae liquid was filtered through a 0.2 μm polycarbonate membrane (Millipore, United States) to remove the particles. Then, the filtrate was regarded as the DOM fraction placed in 1 L conical flask with pre-combusted (450°C, 5 h).

To assess the effects of warming and acidification on the transformation of algae-derived DOM by epiphytic bacteria. Two different temperature (27 and 31°C) and *p*CO_2_ (400 and 1,000 ppm) conditions were used in this work. The following four treatments were set up: (i) 27°C and 400 ppm (low temperature and low carbon, LL), (ii) 27°C and 1,000 ppm (low temperature and high carbon, LH), (iii) 31°C and 400 ppm (high temperature and low carbon, HL), and (iv) 31°C and 1,000 ppm (high temperature and high carbon, HH). *B. pumilus* were grown in above different growth conditions, which were gently bubbled with CO_2_, and the gas-flow rate (0.5 L/min) was controlled using a Bronkhorst mass flow controller. In addition, the initial abundance of *B. pumilus* in each treatment group was approximately (8.51 ± 0.02) × 10^9^ cells L^–1^. All experiments were carried out in triplicate and under dark conditions.

### Excitation-Emission Matrix Combined With Parallel Factor Analysis Modeling

The fluorescence spectrophotometer (Hitachi F-7100, Tokyo, Japan) was used to take three-dimensional fluorescence spectroscopy measurements. The photomultiplier tube’s voltage was set at 700 volts. Excitation (Ex) from 200 to 400 nm and emission (Em) from 250 to 550 nm were identified in successive scanning of fluorescence spectra. The scanning speed was adjusted at 8,000 nm min-1 and the Ex and Em slits were kept at 5 nm. Instrument adjustments were carried out the procedure recommended by the Hitachi F-7100 instruction manual. To eliminate the majority of the Raman scatter, each samples spectroscopy was blank subtracted using ultra-pure water. After that, Raman calibration was performed based on literature (Lawaetz and Stedmon, 2009), and Rayleigh scatter (1st and 2nd order) effects were removed using the manufacturer correction procedure. A PARAFAC analysis was conducted in Matlab 2018b (Mathworks, United States) with the DOMFluor toolbox (Stedmon and Bro, 2008).

Every component model may be tested using a split-half analysis residual analysis, and loadings to provide correct information about fluorescence components. All fluorescence components were described using water Raman units (RU). Fluorescence intensity arbitrary units (a.u.) were utilized to fluorescence peak intensity. Spectroscopic indices were further derived from the EEMs: fluorescence index (FI) is often used to indicate the origin of the DOM. Biological index (BIX) is a measure the degree of autochthonous pollution. The *β/α* (a ratio of two known fluorescing components, i.e., freshness index), and humification index (HIX) showed the humification degree (Parlanti et al., 2000; McKnight et al., 2001).

### Statistical Analysis

The correlation analysis and DOM associated parameters were obtained using the “corrplot” package in RStudio. To rigorously define the significance, the difference was determined significant at the levels of *p* < 0.05, *p* < 0.01, and *p* < 0.001. The measured value of the data is represented by the mean ± standard deviation (SD).

## Results

### Bacterial Abundance, Dissolved Organic Carbon Concentration and pH

**FIGURE 1:**
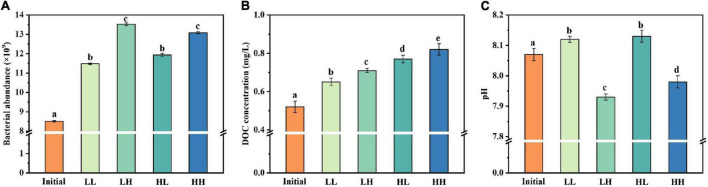
Changes in **(A)** bacterial abundance, **(B)** DOC concentration, and **(C)** pH under warming and acidification conditions. Initial, initial value; LL, 27°C and 400 ppm; LH, 27°C and 1,000 ppm; HL, 31°C and 400 ppm; HH, 31°C and 1,000 ppm. All data are presented as mean ± SD (*n* = 3). Error bars represent the standard error for duplicate cultures. Different letters (e.g., “a,” “b,” “c,” “d,” and “e”) represented significant difference (*p* < 0.05), as determined by a one-way analysis of variance (ANOVA).

The abundance of *B. pumilus* strain growth in different treatment groups is shown in Figure 1. The initial abundance of *B. pumilus* was around (8.51 ± 0.02) × 10^9^ cells L^–1^ in every treatment groups. The *B. pumilus* abundance changed at 30 days in all conditions (Figure 1A). However, there were several differences for the abundance of these treatments. LH and HH achieved higher abundance of up to (13.52 ± 0.07) × 10^9^ and (13.08 ± 0.05) × 10^9^ cells L^–1^, respectively. LL and HL had moderate abundance of (11.48 ± 0.04) × 10^9^ and (11.94 ± 0.08) × 10^9^ cells L^–1^, respectively. This suggests that the significant increase of *B. pumilus* abundance was caused by elevated temperature and *p*CO_2_. The initial DOC concentration was (0.52 ± 0.03) mg L^–1^ for the DOM treatment groups and increased at the end of the 30-day period (Figure 1B). The largest DOC increase occurred in the HH group, which rose by (0.82 ± 0.03) mg L^–1^ DOC over the 30-day period. There were significant differences between each treatment group. The initial pH value of the sample was 8.07 ± 0.02 (Figure 1C). At the end of the experiment, the pH increased in LL and HL groups, conversely, decreased in LH and HH groups as a result of acidification.

### Identification of Bacterial Isolate and Biochemical Characterization

The culture labeled as CA-35 was identified as *Bacillus pumilus* (accession number SRR17041859) based on nucleotide homology and phylogenetic analysis. It was found that the strain CA-35 was Gram-positive rod. Results of various biochemical characteristics of CA-35 was shown in Table 1. The result shows that arabinose, glucose, 7% NaCl, malonate, starch hydrolysis, aesculin, pH 5.7 were positive at the initial phase of the experiment. The individual treatment groups showed different results under different incubation conditions. By contrast, warming and acidification caused changes in some characteristics such as arabinose, glucose, maltose, Voges-Prosk test.

**TABLE 1:** Physiological and biochemical characteristics of *B. pumilus* under warming and acidification conditions.

	Initial	LL	LH	HL	HH
Arabinose	+	−	−	+	−
Glucose	+	−	−	−	+
Maltose	−	−	+	+	−
7% NaCl	+	+	+	+	+
Malonate	+	−	+	−	−
Urease test	−	−	−	−	−
pH 5.7	+	+	+	+	+
Aesculin	+	+	+	+	+
Nitrate reduction	−	−	−	−	−
Starch hydrolysis	+	+	+	+	+
Voges-Prosk	−	+	+	−	−

*A positive reaction is indicated by a plus sign (+), whereas a negative reaction is indicated by a minus sign (−). Initial, initial value; LL, 27°C and 400 ppm; LH, 27°C and 1,000 ppm; HL, 31°C and 400 ppm; HH, 31°C and 1,000 ppm.*

### Fluorescence Components

**FIGURE 2:**
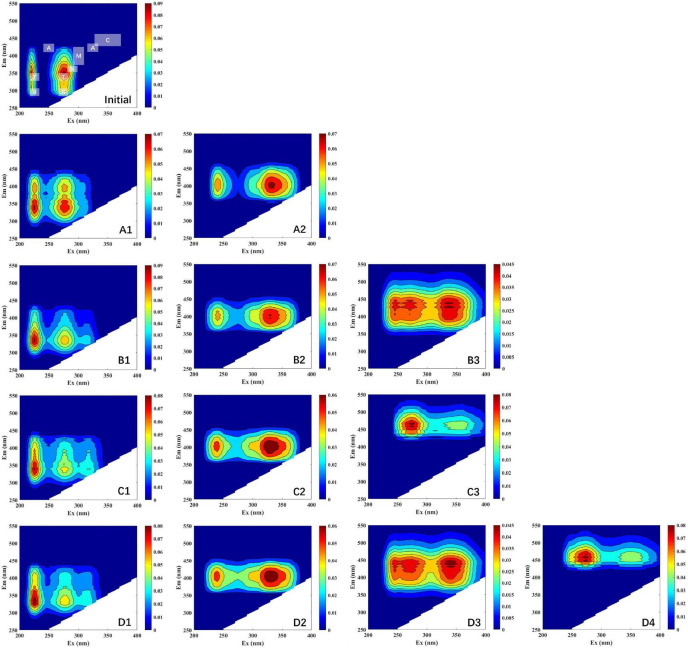
EEM plots of the fluorescence components identified by PARAFAC model. Initial, initial component and intensity; **(A1–A2)**, LL group; **(B1–B3)**, LH group; **(C1–C3)**; HL group; **(D1–D4)**, HH group. Different scales of Raman units (RU) are used to characterize the fluorescence intensity.

All DOM samples collected from the four different treatments were modeled and analyzed with EEM-PARAFAC. In this study, all of the components’ spectral properties were compared to those previously reported in PARAFAC components (Table 2). As shown in Figure 2, one individual component (peak T, tryptophan-like) was identified by the PARAFAC analysis at initial stage. All components (protein-, tryptophan- marine humic-, and humic-like components) in this study have been successfully matched in the OpenFluor database. Component 1 (including A1, B1, C1, and D1; Figure 2) has two fluorescence peaks, located at the Ex/Em wavelength pairs of 225/300–400 and 275/300–400 nm, respectively (Coble, 1996; Coble et al., 1998). Similarly, double peaks have been found in other components. Component 2 (including A2, B2, C2, and D2; Figure 2) was distinguished as marine humic-like components (Ex/Em = 235 and 340/400 nm) (Yao et al., 2011). A humic-like component (peak A, Ex/Em = 250 and 325/425 nm) (Stedmon et al., 2003; Yamashita et al., 2011) associated to terrestrial substances was given to Component 3 (including B3, C3, and D3; Figure 2). Component 4 (including D4; Figure 2) corresponded to humic-like fluorescence (peak C, Ex/Em = 260 and 350/458 nm) (Yamashita and Tanoue, 2008).

**TABLE 2:** EEM fluorescence spectral features attributed to various organic matter sources.

Traditional peak	Ex/Em	Description	Probable origin	References
Peak B	225/305 275/305	Protein-like	Autochthonous tyrosine-like fluorescence	Coble et al., 1998; Para et al., 2010
Peak T	225/330–340 275/330–340	Protein-like Tryptophan-like	Autochthonous	Coble, 1996; Coble et al., 1998
Peak N	280/360–370	Unknown	Unknown	Coble et al., 1998
Peak A	250/425 325/425	Humic-like	Terrestrial	Stedmon et al., 2003; Yamashita et al., 2011
Peak M	290–310/370–420	Humic-like	Microbial processing of organic matter	Murphy et al., 2008
Peak C	320–360/430–460	Humic-like	Terrestrial/Autochthonous	Murphy et al., 2008

### Fluorescence Indices and Peaks

**FIGURE 3:**
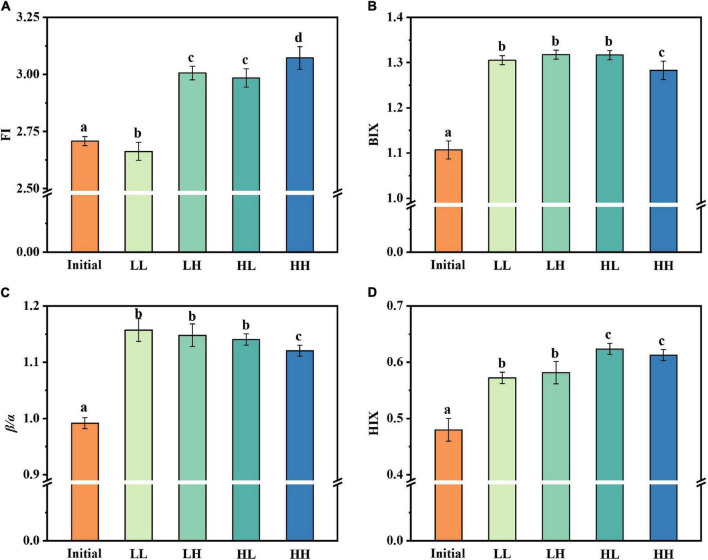
Changes in fluorescence indices under warming and acidification conditions. **(A–D)** Represent FI, BIX, β/α, and HIX respectively. Initial, initial value; LL, 27°C and 400 ppm; LH, 27°C and 1,000 ppm; HL, 31°C and 400 ppm; HH, 31°C and 1000 ppm. All data are presented as mean ± SD (*n* = 3). Error bars represent the standard error for duplicate cultures. Different letters (e.g., “a,” “b,” “c,” and “d”) represented significant difference (*p* < 0.05), as determined by a one-way analysis of variance (ANOVA).

The fluorescence index was further used as a parameter indicating changes in fluorescence characteristics (Figure 3). The origin of DOM is identified *via* FI. The three treatment groups had similar values for FI, which had no obvious change under warming and acidifying conditions. The freshness index was also calculated, with the β peak representing newly produced DOM and the α peak representing more decomposed DOM. All of the samples had β/α values between 1.12 and 1.16, whereas LL had maximum values of 1.16. For BIX, the values are almost appropriate (i.e., 1.28–1.32). The HIX is an indicator of how much organic matter has degraded. There are varying degrees of alteration in the degree of humification, especially in HH group.

**FIGURE 4:**
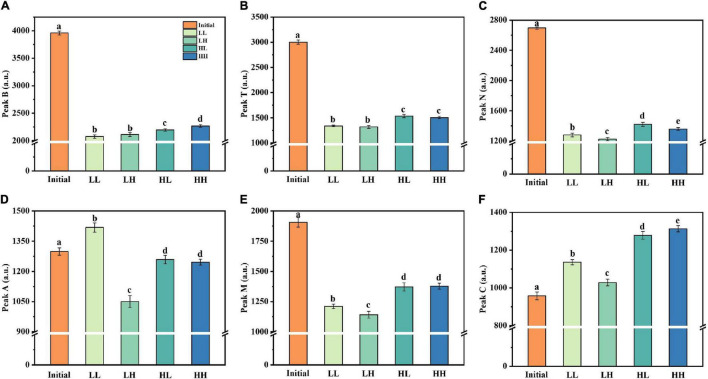
Changes in fluorescence peaks (peaks B, T, N, A, M, and C) under warming and acidification conditions. **(A–F)** Represent peak B, peak T, peak N, peak A, peak M, and peak C respectively. Initial, initial value; LL, 27°C and 400 ppm; LH, 27°C and 1,000 ppm; HL, 31°C and 400 ppm; HH, 31°C and 1,000 ppm. All data are presented as mean ± SD (*n* = 3). Error bars represent the standard error for duplicate cultures. Different letters (e.g., “a,” “b,” “c,” “d,” and “e”) represented significant difference (*p* < 0.05), as determined by a one-way analysis of variance (ANOVA).

The fluorescence intensity of the peaks was altered in all treatment groups after 30 days of incubation (Figure 4). Peak B (protein-like) and peak T (protein-like) maintained a high fluorescence intensity (3,000–4,000 a.u.) in the initial phases, with a similar degree of variation for treatment groups. There was minimal fluorescence intensity in the LL group, suggesting that warming and acidification slowed bacterial consumption of protein-like substances. Additionally, peak N, an unknown component, followed a similar trend to peaks B and T, with generally lower values for all treatment groups. For peak A, acidification resulted in a reduction in the fluorescence intensity of the like-humic substances (i.e., LH and HH groups). However, the fluorescence intensity of peak M (like-humic) decreased and the warming slows down the utilization efficiency of peak M (i.e., HL and HH groups). The only difference is that the fluorescence intensity of peak C (like-humic) is generally increased in all groups.

## Discussion

Phytoplankton-DOM (especially autochthonous DOM) is a highly more bioavailable and high-molecular-weight DOM (Hama and Yanagi, 2001). Heterotrophic bacteria prefer phytoplankton-derived DOM over terrestrial DOM as a substrate for catabolic activities when both are present (Kritzberg et al., 2004). A number of previous cultivation experiments have studied the dynamics of phytoplankton-derived DOM observed with carbon and nutrient additions (Romera-Castillo et al., 2010; Goto et al., 2017, 2020). It has also been shown that phytoplankton produce different characteristics of DOM under different conditions (Liu et al., 2021a,c). In order to better investigate the transformation of DOM by *B. pumilus*, *S. dohrnii*-derived DOM was used as the sole carbon sources. In addition, previously published data demonstrated that the characteristics of DOM produced by *S. dohrnii* are relatively single (Liu et al., 2021a,b). In this study, we have isolated and identified the epiphytic bacteria (*B. pumilus*) of *S. dohrnii*. We collected the *S. dohrnii*-derived DOM in the degradation, because of its enriched fluorescence characteristics and high fluorescence intensity. The results showed that *S. dohrnii*-derived DOM fluorescence is predominantly protein-like, which gradually becomes more and more humic-like within 30 days. This suggests that the *B. pumilus* transformed *S. dohrnii*-derived DOM into more complex substance under increasing temperature and acidification. Together, the present study highlights that warming and acidification affect the effectiveness of bacterial transformation of DOM.

### Regulation of Phytoplankton-Derived Dissolved Organic Matter by Bacteria

According to Boyce et al. (2010) reported, total phytoplankton abundance is rapidly declining as a result of warming, with diatoms being the most impacted category (Toseland et al., 2013). Under stressed conditions like as warming, acidification, and nutrient scarcity, phytoplankton releases large amounts of DOM. Bacterial abundance was inversely proportional to phytoplankton abundance under these circumstances. Warming has been demonstrated to favor bacterial growing, which might be owing to two factors: (1) the favorable effect of temperature on bacteria, with more bacterial cells at higher temperatures (Apple et al., 2006), and (2) the availability of more enriched DOM for bacteria. Put simply, phytoplankton produces labile DOC, which heterotrophic bacteria can convert to recalcitrant DOC (Stoderegger and Herndl, 1998). Previous investigations have shown that the addition of *p*CO_2_ had considerable impact on overall bacterial abundance (Baragi and Anil, 2016). However, in this study, algae-DOM was employed as the only carbon source and the LH group showed a relative increase in bacterial abundance. This is likely because high *p*CO_2_ only slowed the rate of increase in bacterial abundance, but did not limit/inhibit it. Besides, the increase in *B. pumilus* abundance might be attributed to the effect of decreased pH, which increases respiration, as a result, the metabolic and energy cost of bacteria (Siu et al., 2014).

In the present study, a fixed amount of *S. dohrnii*-derived DOM was added at the initial phase. A considerable portion of the bioavailable fraction of DOM was reduced by bacterial respiration and absorption. However, the DOC concentration showed a distinct increase, suggesting that DOM components were transformed by bacteria to produce new DOC (i.e., production was higher than consumption) under warming and acidification. The DOM can be used as a substrate for remineralization of carbon by heterotrophic microorganisms. Microorganisms use enzymes to catalyze DOC into smaller molecules that can be transported to the environment through bacterial cell membranes (Arnosti, 2011). The organic carbon is subsequently absorbed into the biomass or expelled as DOC as metabolic products (Arnosti, 2011). This provides a new perspective for observing the transformation process of diatoms-derived DOM by epiphytic bacteria.

### Excitation-Emission Matrix Combined With Parallel Factor Analysis Components

To further understand the characteristics of DOM fluorophores, we built models with components and used a split-half analysis to validate them all. Four fluorescent components, including typically occurring protein- and humic-like components, were detected in *S. dohrnii*-derived DOM samples (Figure 2). At the initial phase, protein-like components (peak B and T) were detected. For example, peak T is typically connected to autochthonous tryptophan-like compounds, amino acids, and proteins. In aquatic ecosystems, peak T usually represents the proteinaceous material produced by phytoplankton and bacteria. Moreover, the presence of visible components (peak T) could be attributed by energy transfer to tryptophan and quenching by adjacent groups suppressing tyrosine fluorescence (Lakowicz, 2013). Some of the components have previously been related to high molecular weight and aromatic humic material characterized as peaks A, M, and C in the literature (Wünsch et al., 2015).

All treatment groups essentially produced two-peak patter dominated by discrete *S. dohrnii*-derived DOM fluorophores, indicating that different compounds, such as protein- and humic-like have been described in the algae-DOM. Several studies have shown that protein peaks (e.g., tryptophan-like) are dominant in phytoplankton growth (Stedmon and Markager, 2005; Jørgensen et al., 2011; Liu et al., 2021b). In previous studies (Kinsey et al., 2018; Liu et al., 2021a), DOM produced in phytoplankton showed similar fluorescence patterns to presented here. As reported by Fukuzaki et al. (2014), the DOM spectra of algal cultures have also been studied. Although the DOM fluorophores are different, the DOM patterns produced are generally similar. The current results show that bacteria use and transform DOM to obtain different fluorescent components. Under elevated temperature and acidified conditions, more fluorescent components were identified, indicating that certain physicochemical characteristics of the bacteria were affected (Table 1), further leading to altered DOM utilization by the bacteria and more humic substances being produced.

In the current study, results showed that the fluorescence values of peak C increased on the whole, while peaks B, T, N, A, and M decreased comparably to the initial value. It suggested that the epiphytic bacteria utilize DOM, which leads to the increase of bacterial abundance, and then the DOM is transformed into recalcitrant DOM and gradual accumulation by epiphytic bacteria. It is noteworthy that on the time scale of deep ocean circulation, these humus-like substances have proven to be recalcitrant, whereas only a small fraction of humic-like substances were biodegradable (Catalá et al., 2015). Besides, the generally low fluorescence in the open ocean suggests that the degradation experiments were conducted over a longer period time than our and other studies (Gruber et al., 2006; Fukuzaki et al., 2014). Indeed, a prior study found that a single strains is incapable of entirely degrading high molecular weight DOM compounds, meaning that multiple bacterial species must work together (Horemans et al., 2013). Briefly, the utilization and transformation of DOM by a single bacterium take much longer.

The spectroscopic index provided a clear insight into the process of DOM-related substance changes. The values of FI were all greater than 1.8, demonstrating that bacteria were primarily responsible for the fluorescence component of DOM transformation and production (Lavonen et al., 2015). In aquatic ecosystems, BIX could be used as a measurement of DOM traceability, with larger values indicating more DOM degradation. The significant changes in β/α and BIX values over a short period of time indicated that endogenous carbon products are most likely produced through bacterial processing of DOM. The HIX indicates the degree of degradation of organic matter, with higher values characteristic of higher molecular weight, aromatic compounds. In other words, the humic concentration of DOM is roughly proportional to HIX (Huguet et al., 2009).

Elevated temperature and acidification caused a general increase in FI and β/α values. Interestingly, we found that β/α values decreased in the HH group, which may be due to the fact that the highly decomposed DOM was higher than recently derived DOM. Besides, both warming and acidification led to a decrease in HIX value. From the current results, the effect of acidification was more prominent. However, the BIX value of each group was not significantly different.

### Transformations and Connections of Organic Matter

The overall DOM concentration increased by 9.23–26.15% in this study due to elevated temperature and acidification. This result indicates that warming promoted the accumulation of microbial-derived DOM and depressed the aromaticity of DOM. Previous studies have demonstrated that microbial-derived aliphatic carbon is the most labile DOM, which is more readily absorbed than plant-derived aromatic carbon (Yang et al., 2016). Changes in bacterial abundance and DOM properties such as pH, fluorophores, and fluorescence intensity could be explain the discrepancy. Acidification is linked to the decomposition of more refractory organic carbon (e.g., lignocelluloses), as compared to warming (Williamson et al., 1999). Specifically, pH increased the aromatic fractions in DOM (peak C), which are recognized as organic matter produced by bacteria. The bacterial abundance in our study was increased from 8.51 × 10^9^ to 13.52 × 10^9^ cells L^–1^ under acidification conditions, besides, significant increase in DOC concentration. Thus, it is regarded as enhanced utilization of aromatic DOM by bacteria.

**FIGURE 5:**
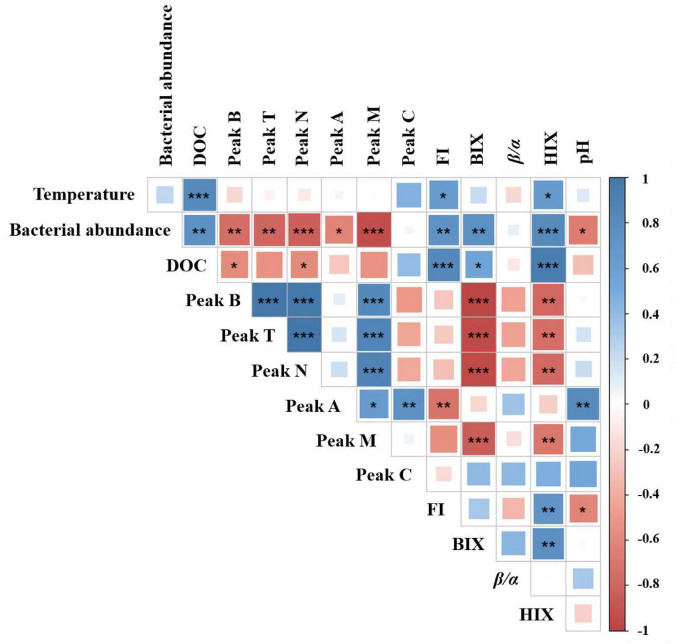
Correlation analysis between DOM-associated parameters and temperature and pH potential impact. Pearson’s correlation coefficients are shown by square colors and sizes. *, **, and *** represent significance degrees at *p* < 0.05, *p* < 0.01, and *p* < 0.001, respectively. A positive connection is represented by blue, whereas a negative correlation is shown by red.

Pearson’s correlation analysis showed significant variability between warming and acidification for DOM-associated parameters. As shown in Figure 5, temperature and DOC concentration had a significant positive correlation (*p* < 0.001). Besides, FI and HIX were positively correlated with temperature (*p* < 0.05), respectively. These results indicating that temperature affects microbial activity and production, which was consistent with results from previous studies (Thangaraj and Sun, 2021). The pH and peak A had a significant positive correlation (*p* < 0.01), and bacteria abundance and FI were significantly negatively correlated with pH (*p* < 0.05). Moreover, the formation of aromatic DOM or humic-like is thought to be facilitated by acidification-driven microorganisms. This appears to be corroborated by the fact that when the temperature rises, and pH decreases (0.09–0.14 units). Bacterial abundance and microbial activity have both been shown to increase when pH is raised (Han et al., 2022). This is overall consistent with our results.

As discussed above, elevated temperature and acidification showed different impacts on DOM utilization and transform by bacteria. This work has important implications for better understanding how microbes in the ocean transform and sequester DOM. Our findings show that warming and acidification conditions will exacerbate the accumulation of organic matter, particularly phytoplankton-derived aromatic substances. Given the crucial impacts and consequences of bacteria on the use of phytoplankton-DOM under warming and acidification conditions, further investigations regarding carbon transform and release process in natural environments (especially microbial environment) under global climate change deserves serious attention.

## Conclusion

This work provided direct evidences for bacterial utilization and transformation of phytoplankton-DOM under warming and acidification conditions. Our findings emphasized that warming reduced the efficiency of bacterial use of protein-like substances, while acidification promoted the transformation of humus-like substances (e.g., flavins and phenols, etc.). The simultaneous increment of warming and acidification accelerates the accumulation of recalcitrant DOM. Together, temperature and pH have important influence in the chemical composition and remineralization of DOM in marine microorganisms.

## Data Availability Statement

The original contributions presented in the study are included in the article/supplementary material, further inquiries can be directed to the corresponding author/s.

## Author Contributions

JS and YL contributed to conception and design of the study. YL and XW organized the database. YL performed the statistical analysis and wrote the first draft of the manuscript. All authors contributed to manuscript revision, read, and approved the submitted version.

## Conflict of Interest

The authors declare that the research was conducted in the absence of any commercial or financial relationships that could be construed as a potential conflict of interest.

## Publisher’s Note

All claims expressed in this article are solely those of the authors and do not necessarily represent those of their affiliated organizations, or those of the publisher, the editors and the reviewers. Any product that may be evaluated in this article, or claim that may be made by its manufacturer, is not guaranteed or endorsed by the publisher.

## References

[B1] Apple J. K. Del Giorgio P. Kemp W. M. (2006). Temperature regulation of bacterial production, respiration, and growth efficiency in a temperate salt-marsh estuary. *Aquat. Microb. Ecol.* 43:243. 10.3354/ame043243

[B2] Arnosti C. (2011). Microbial extracellular enzymes and the marine carbon cycle. *Annu. Rev. Mar. Sci.* 3 401–425. 10.1146/annurev-marine-120709-142731 21329211

[B3] Baragi L. V. Anil A. C. (2016). Synergistic effect of elevated temperature, *p*CO2 and nutrients on marine biofilm. *Mar. Pollut. Bull.* 105 102–109. 10.1016/j.marpolbul.2016.02.049 26936123

[B4] Boyce D. G. Lewis M. R. Worm B. (2010). Global phytoplankton decline over the past century. *Nature* 466 591–596. 10.1038/nature09268 20671703

[B5] Catalá T. S. Reche I. Fuentes-Lema A. Romera-Castillo C. Nieto-Cid M. Ortega-Retuerta E. (2015). Turnover time of fluorescent dissolved organic matter in the dark global ocean. *Nat. Commun.* 6:5986. 10.1038/ncomms6986 25631682

[B6] Coble P. G. (1996). Characterization of marine and terrestrial DOM in seawater using excitation-emission matrix spectroscopy. *Mar. Chem.* 51 325–346. 10.1016/0304-4203(95)00062-3

[B7] Coble P. G. Del Castillo C. E. Avril B. (1998). Distribution and optical properties of CDOM in the Arabian Sea during the 1995 Southwest Monsoon. *Deep Sea Res. Part II* 45 2195–2223. 10.1016/S0967-0645(98)00068-X

[B8] Del Giorgio P. A. Duarte C. M. (2002). Respiration in the open ocean. *Nature* 420 379–384. 10.1038/nature01165 12459775

[B9] Falkowski P. G. Katz M. E. Knoll A. H. Quigg A. Raven J. A. Schofield O. (2004). The evolution of modern eukaryotic phytoplankton. *Science* 305 354–360. 10.1126/science.1095964 15256663

[B10] Fuhrman J. A. (2009). Microbial community structure and its functional implications. *Nature* 459 193–199. 10.1038/nature08058 19444205

[B11] Fukuzaki K. Imai I. Fukushima K. Ishii K. I. Sawayama S. Yoshioka T. (2014). Fluorescent characteristics of dissolved organic matter produced by bloom-forming coastal phytoplankton. *J. Plankton Res.* 36, 685–694. 10.1093/plankt/fbu015

[B12] Goto S. Tada Y. Suzuki K. Yamashita Y. (2017). Production and reutilization of fluorescent dissolved organic matter by a marine bacterial strain, *Alteromonas macleodii*. *Front. Microbiol.* 8:507. 10.3389/fmicb.2017.00507 28400762 PMC5368175

[B13] Goto S. Tada Y. Suzuki K. Yamashita Y. (2020). Evaluation of the production of dissolved organic matter by three marine bacterial strains. *Front. Microbiol.* 11:2553. 10.3389/fmicb.2020.584419 33178167 PMC7593260

[B14] Grossart H. P. Allgaier M. Passow U. Riebesell U. (2006). Testing the effect of CO_2_ concentration on the dynamics of marine heterotrophic bacterioplankton. *Limnol. Oceanogr.* 51 1–11. 10.4319/lo.2006.51.1.0001

[B15] Gruber D. F. Simjouw J. P. Seitzinger S. P. Taghon G. L. (2006). Dynamics and characterization of refractory dissolved organic matter produced by a pure bacterial culture in an experimental predator-prey system. *Appl. Environ. Microbiol.* 72 4184–4191. 10.1128/AEM.02882-05 16751530 PMC1489638

[B16] Hama T. Yanagi K. (2001). Production and neutral aldose composition of dissolved carbohydrates excreted by natural marine phytoplankton populations. *Limnol. Oceanogr.* 46 1945–1955. 10.4319/lo.2001.46.8.1945

[B17] Han Y. Qu C. Hu X. Wang P. Wan D. Cai P. (2022). Warming and humidification mediated changes of DOM composition in an Alfisol. *Sci. Total Environ.* 805:150198. 10.1016/j.scitotenv.2021.150198 34537712

[B18] Hansell D. A. Carlson C. A. Schlitzer R. (2012). Net removal of major marine dissolved organic carbon fractions in the subsurface ocean. *Glob. Biogeochem. Cycles* 26:GB1016. 10.1029/2011GB004069

[B19] Hansell D. A. Carlson C. A. Repeta D. J. Schlitzer R. (2009). Dissolved organic matter in the ocean: a controversy stimulates new insights. *Oceanography* 22 202–211. 10.5670/oceanog.2009.109

[B20] Horemans B. Vandermaesen J. Smolders E. Springael D. (2013). Cooperative dissolved organic carbon assimilation by a linuron-degrading bacterial consortium. *FEMS Microbiol. Ecol.* 84 35–46. 10.1111/1574-6941.12036 23106441

[B21] Huguet A. Vacher L. Relexans S. Saubusse S. Froidefond J. M. Parlanti E. (2009). Properties of fluorescent dissolved organic matter in the Gironde Estuary. *Org. Geochem.* 40 706–719. 10.1016/j.orggeochem.2009.03.002

[B22] Jørgensen L. Stedmon C. A. Kragh T. Markager S. Middelboe M. Søndergaard M. (2011). Global trends in the fluorescence characteristics and distribution of marine dissolved organic matter. *Mar. Chem.* 126 139–148. 10.1016/j.marchem.2011.05.002

[B23] Kinsey J. D. Corradino G. Ziervogel K. Schnetzer A. Osburn C. L. (2018). Formation of chromophoric dissolved organic matter by bacterial degradation of phytoplankton-derived aggregates. *Front. Mar. Sci.* 4:430. 10.3389/fmars.2017.00430

[B24] Kritzberg E. S. Cole J. J. Pace M. L. Granéli W. Bade D. L. (2004). Autochthonous versus allochthonous carbon sources of bacteria: results from whole-lake ^13^C addition experiments. *Limnol. Oceanogr.* 49 588–596. 10.4319/lo.2004.49.2.0588

[B25] Lakowicz J. R. (2013). *Principles of Fluorescence Spectroscopy.* Berlin: Springer Science and Business Media.

[B26] Lavonen E. E. Kothawala D. N. Tranvik L. J. Gonsior M. Schmitt-Kopplin P. Köhler S. J. (2015). Tracking changes in the optical properties and molecular composition of dissolved organic matter during drinking water production. *Water Res.* 85, 286–294. 10.1016/j.watres.2015.08.024 26342182

[B27] Lawaetz A. J. Stedmon C. A. (2009). Fluorescence intensity calibration using the Raman scatter peak of water. *Appl. Spectrosc.* 63 936–940. 10.1366/000370209788964548 19678992

[B28] Lin H. Xu H. Cai Y. Belzile C. Macdonald R. W. Guo L. (2021). Dynamic changes in size-fractionated dissolved organic matter composition in a seasonally ice-covered Arctic River. *Limnol. Oceanogr.* 66 3085–3099. 10.1002/lno.11862

[B29] Liu Y. Kan J. He C. Shi Q. Liu Y. X. Fan Z. C. (2021a). Epiphytic bacteria are essential for the production and transformation of algae-derived carboxyl-Rich alicyclic molecule (CRAM)-like DOM. *Microbiol. Spectrum* 9:e0153121. 10.1128/Spectrum.01531-21 34668747 PMC8528127

[B30] Liu Y. Sun J. Wang X. Liu X. Wu X. Chen Z. (2021d). Fluorescence characteristics of chromophoric dissolved organic matter in the Eastern Indian Ocean: a case study of three subregions. *Front. Mar. Sci.* 8:742595. 10.3389/fmars.2021.742595

[B31] Liu Y. Liu X. Sun J. (2021c). Response of chlorophyll fluorescence characteristics and dissolved organic matter for marine diatom *Skeletonema dohrnii* under stress from penicillin and Zn^2+^. *Plants* 10:2684. 10.3390/plants10122684 34961155 PMC8708301

[B32] Liu Y. Kan J. Yang J. Noman M. A. Sun J. (2021b). Bacterial community composition and chromophoric dissolved organic matter differs with culture time of *Skeletonema dohrnii*. *Diversity* 13:150. 10.3390/d13040150

[B33] McKnight D. Boyer E. Westerhoff P. K. Doran P. T. Kulbe T. Andersen D. (2001). Spectrofluorometric characterization of dissolved organic matter for indication of precursor organic material and aromaticity. *Limnol. Oceanogr.* 46 38–48. 10.4319/lo.2001.46.1.0038

[B34] Meehl G. A. Stocker T. F. Collins W. D. Friedlingstein P. Gaye T. Gregory J. M. (2007). “Global climate projections,” in *Climate Change 2007: The Physical Science Basis. Contribution of Working Group I to the Fourth Assessment Report of the Intergovernmental Panel on Climate Change*, eds Solomon S. Qin D. Manning M. Chen Z. Marquis M. Averyt K. B. (Cambridge: Cambridge University Press).

[B35] Moens F. Weckx S. De Vuyst L. (2016). Bifidobacterial inulin-type fructan degradation capacity determines cross-feeding interactions between bifidobacteria and *Faecalibacterium prausnitzii*. *Int. J. Food Microbiol.* 231 76–85. 10.1016/j.ijfoodmicro.2016.05.015 27233082

[B36] Murphy K. R. Stedmon C. A. Waite T. D. Ruiz G. M. (2008). Distinguishing between terrestrial and autochthonous organic matter sources in marine environments using fluorescence spectroscopy. *Mar. Chem*. 108 40–58. 10.1016/j.marchem.2007.10.003

[B37] Pachauri R. K. Allen M. R. Barros V. R. Broome J. Cramer W. Christ R. (2014). *Climate Change 2014: Synthesis Report. In Contribution of Working Groups I, II and III to the Fifth Assessment Report of the Intergovernmental Panel on Climate Change.* Geneva: IPCC.

[B38] Para J. Coble P. G. Charrìère B. Tedetti M. Fontana C. Sempere R. (2010). Fluorescence and absorption properties of chromophoric dissolved organic matter (CDOM) in coastal surface waters of the northwestern Mediterranean Sea, influence of the Rhône River. *Biogeosciences* 7 4083–4103. 10.5194/bg-7-4083-2010

[B39] Parlanti E. Wörz K. Geoffroy L. Lamotte M. (2000). Dissolved organic matter fluorescence spectroscopy as a tool to estimate biological activity in a coastal zone submitted to anthropogenic inputs. *Org. Geochem.* 31 1765–1781. 10.1016/S0146-6380(00)00124-8

[B40] Pomeroy L. R. Williams P. J. I. Azam F. Hobbie J. E. (2007). The microbial loop. *Oceanography* 20 28–33. 10.5670/oceanog.2007.45

[B41] Riebesell U. Aberle-Malzahn N. Achterberg E. P. Algueró-Muñiz M. Alvarez-Fernandez S. Arístegui J. (2018). Toxic algal bloom induced by ocean acidification disrupts the pelagic food web. *Nat. Clim. Change* 8 1082–1086. 10.1038/s41558-018-0344-1

[B42] Rodríguez-Vidal F. J. García-Valverde M. Ortega-Azabache B. González-Martínez Á Bellido-Fernández A. (2020). Characterization of urban and industrial wastewaters using excitation-emission matrix (EEM) fluorescence: searching for specific fingerprints. *J. Environ. Manage.* 263:110396. 10.1016/j.jenvman.2020.110396 32174533

[B43] Romera-Castillo C. Sarmento H. Álvarez-Salgado X. A. Gasol J. M. Marrasé C. (2010). Production of chromophoric dissolved organic matter by marine phytoplankton. *Limnol. Oceanogr.* 55 446–454. 10.4319/lo.2010.55.1.0446

[B44] Siu N. Apple J. K. Moyer C. L. (2014). The effects of ocean acidity and elevated temperature on bacterioplankton community structure and metabolism. *Open J. Ecol.* 4:434. 10.4236/oje.2014.48038

[B45] Stedmon C. A. Bro R. (2008). Characterizing dissolved organic matter fluorescence with parallel factor analysis: a tutorial. *Limnol. Oceanogr. Methods* 6 572–579. 10.4319/lom.2008.6.572

[B46] Stedmon C. A. Markager S. (2005). Tracing the production and degradation of autochthonous fractions of dissolved organic matter by fluorescence analysis. *Limnol. Oceanogr.* 50 1415–1426. 10.4319/lo.2005.50.5.1415

[B47] Stedmon C. A. Markager S. Bro R. (2003). Tracing dissolved organic matter in aquatic environments using a new approach to fluorescence spectroscopy. *Marine Chem.* 82, 239–254. 10.1016/S0304-4203(03)00072-0

[B48] Stocker T. Qin D. Plattner G. Tignor M. Allen S. Boschung J. (2014). *Climate Change 2013: The Physical Science Basis: Working Group I Contribution to the Fifth Assessment Report of the Intergovernmental Panel on Climate Change.* Cambridge: Cambridge university press.

[B49] Stoderegger K. E. Herndl G. J. (1998). Production and release of bacterial capsular material and its subsequent utilization by marine bacterioplankton. *Limnol. Oceanogr.* 43 877–884. 10.4319/lo.1998.43.5.0877

[B50] Thangaraj S. Sun J. (2020). The biotechnological potential of the marine diatom *Skeletonema dohrnii* to the elevated temperature and *p*CO_2_ concentration. *Mar. Drugs* 18:259. 10.3390/md18050259 32429035 PMC7281586

[B51] Thangaraj S. Sun J. (2021). Transcriptomic reprogramming of the oceanic diatom *Skeletonema dohrnii* under warming ocean and acidification. *Environ. Microbiol.* 23 980–995. 10.1111/1462-2920.15248 32975013

[B52] Thornton D. C. (2014). Dissolved organic matter (DOM) release by phytoplankton in the contemporary and future ocean. *Eur. J. Phycol.* 49 20–46. 10.1080/09670262.2013.875596

[B53] Toseland A. Daines S. J. Clark J. R. Kirkham A. Strauss J. Uhlig C. (2013). The impact of temperature on marine phytoplankton resource allocation and metabolism. *Nat. Clim. Chang.* 3 979–984. 10.1038/nclimate1989

[B54] Williamson C. E. Morris D. P. Pace M. L. Olson O. G. (1999). Dissolved organic carbon and nutrients as regulators of lake ecosystems: resurrection of a more integrated paradigm. *Limnol. Oceanogr.* 44 795–803. 10.4319/lo.1999.44.3_part_2.0795

[B55] Wünsch U. J. Murphy K. R. Stedmon C. A. (2015). Fluorescence quantum yields of natural organic matter and organic compounds: implications for the fluorescence-based interpretation of organic matter composition. *Front. Mar. Sci.* 2:98. 10.3389/fmars.2015.00098

[B56] Yamashita Y. Tanoue E. (2008). Production of bio-refractory fluorescent dissolved organic matter in the ocean interior. *Nat. Geosci.* 1 579–582. 10.1038/ngeo279

[B57] Yamashita Y. Panton A. Mahaffey C. Jaffé R. (2011). Assessing the spatial and temporal variability of dissolved organic matter in Liverpool Bay using excitation-emission matrix fluorescence and parallel factor analysis. *Ocean Dyn.* 61 569–579. 10.1007/s10236-010-0365-4

[B58] Yang Z. Wullschleger S. D. Liang L. Graham D. E. Gu B. (2016). Effects of warming on the degradation and production of low molecular-weight labile organic carbon in an Arctic tundra soil. *Soil Biol. Biochem.* 95 202–211. 10.1016/j.soilbio.2015.12.022

[B59] Yao X. Zhang Y. Zhu G. Qin B. Feng L. Cai L. (2011). Resolving the variability of CDOM fluorescence to differentiate the sources and fate of DOM in Lake Taihu and its tributaries. *Chemosphere* 82 145–155. 10.1016/j.chemosphere.2010.10.049 21071060

